# Rectal large cell neuroendocrine carcinoma

**DOI:** 10.1093/omcr/omaf185

**Published:** 2025-09-28

**Authors:** Panagiotis Alexandros Drakos, Antonia A Prountzopoulou, Efrossini Totskas, Konstantinos Stamou

**Affiliations:** Barts and the London School of Medicine and Dentistry - Queen Mary, University of London, Garrod Building, Queen Mary University of London, London E1 2AD, England, United Kingdom; 2nd Department of Surgery, MITERA Hospital, 6 Erythrou Stavrou Street & Kifisias Avenue, Marousi, Athens, Attica 15123, Greece; University Medical Center Groningen, Faculty of Medical Sciences, Hanzeplein 1, 9713 GZ Groningen, Province of Groningen, Netherlands; 2nd Department of Surgery, MITERA Hospital, 6 Erythrou Stavrou Street & Kifisias Avenue, Marousi, Athens, Attica 15123, Greece

**Keywords:** oncology, gastroenterology, endocrinology and metabolism

## Abstract

Rectal large cell neuroendocrine carcinoma (LCNEC) is an exceedingly rare and aggressive neoplasm with a poor prognosis and median survival of 4–16 months. Diagnosis is challenging due to the clinical overlap with classical colorectal adenocarcinoma, and accurate diagnosis is reliant on histological examination via immunohistochemistry (IHC). For the diagnosis of LCNEC, neuroendocrine markers such as Synaptophysin, CD56, chromogranin A and Ki-67 are major determinants of the disease. We present a double case report of two individuals initially assumed diagnosed as rectal adenocarcinoma who were then re-diagnosed with rectal LCNEC via post-surgical IHC. Both patients received neo-adjuvant chemotherapy yet still developed metastatic disease. This report intends to appraise the role of routine early IHC as a critical tool for diagnosis and to guide management planning. Given the rarity and volatility of rectal LCNEC, further research is desperately needed to develop tailored treatment measures and improve patient outcomes.

## Introduction

Neuroendocrine tumours (NETs) represent an increasingly recognised and diverse group of gastrointestinal neoplasms. Rectal NET (r-NET) incidence was once considered lower; however, the development of endoscopic technologies and regular screening has increased diagnoses [1, 2]. These neoplasms arise from neuroendocrine cells found amply in the gut, yet represent only 2% of gastrointestinal malignancies [3]. Accurate classification of NETs is critical to establish management plans and is predicated on histological analysis, (particularly mitotic rate and Ki-67 index) which places NETs on a G1-G3 grading system based on proliferative capacity [3–5]. Based on this system, r-NETs encompass the well-differentiated neoplasms in the G1-G2 category [3].

However, neuroendocrine carcinomas (NECs) represent a poorly differentiated, higher-grade NET in the G3 category [3]. Summarily, the combined usage of the G#, classical TNM and immunohistochemical markers is imperative in making a diagnosis. Specifically, positive staining for neuroendocrine markers is pertinent: synaptophysin, chromogranin A, Ki-67, etc [4]. Within this category, exists the rectal large-cell neuroendocrine carcinoma (LCNEC). Rectal LCNEC are a sufficiently rare subset of r-NECs characterised by large-cell morphology, specific histological patterns such as ample cytoplasm and frequent nucleoli, and necrotic regions [3, 4]. This subset of r-NETs accounts for 0.25% of all colorectal cancers and is associated with an expected survival of 4–16 months [5].

This double case report discusses two separate patients with rectal LCNEC and reiterates the clinical significance of rigorous immunohistochemistry (IHC) staining protocol.

## Case report

**Figure 1:**
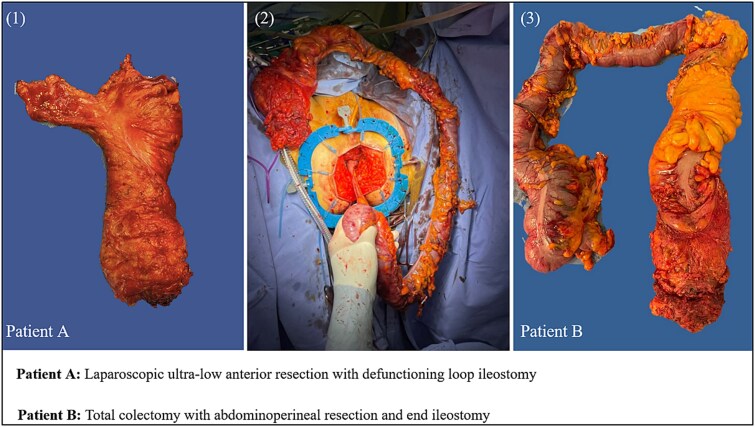
Resected specimen from patient A and B. Figure 1: Image 1 indicates the resected specimen from patient A. image 2 indicates the resection process from patient A. image 3 indicates the resected specimen of patient B.

Patient A was a 53-year-old male who presented with blood in stool; endoscopy showed a low-rectal adenocarcinoma, diagnosed via standard eosin-haematoxylin stain. Initial staging with MRI was cT3N1M0. He was treated with neo-adjuvant long course chemoradiotherapy (LcCRT) with 54 Gy and capecitabine. 8 weeks after completing radiotherapy, MRI and rectoscopy showed no substantial clinical response. Furthermore, he underwent laparoscopic ultra-low anterior resection with defunctioning loop ileostomy (Fig. 1). Histological examination indicated an ulcerative poorly differentiated LCNEC with partial response to previous treatment (TRG 2). Additionally, IHC showed Synaptophysin [+], CD56 [+], Chromogranin A [−] and Ki67: 80% and final staging was ypT3N1aM0 (Table 1). The patient was given adjuvant FOLFOX chemotherapy (folinic acid, fluorouracil and oxaliplatin). Due to lack of response, the regimen was changed to carboplatin-etoposide. The patient died of metastatic disease 11 months postoperatively.

**Table 1:** Patient A/B biopsy results.

	Patient A	Patient B
Synaptophysin	Positive (+)	Positive (+)
CD56	Positive (+)	Positive (+)
Chromogranin A	Negative (−)	‘Mostly’ Negative (−)
Tumor Regression Grade (TRG)	TRG 2	TRG 2
Lymph Nodes	1/51	7/77
Perineural Invasion (PNi)	Positive (+)	Positive (+)
Lymphatic Invasion (Lyi)	Positive (+)	Positive (+)
Vascular Invasion (Vi)	Positive (+)	Positive (+)
Circumferential Resection Margin (CRM)	Negative (−)	Positive (+)
Mismatch Repair (MMR) Status	Proficient	Proficient

Table 1 summarizes the core biopsy/histological results for Patient A and Patient B.

Patient B was a 67-year-old female with a history of ulcerative colitis; subsequently endoscopy, rectal MRI and eosin-haematoxylin staining showed low-rectal adenocarcinoma cT3N1M0 with a threatening circumferential resection margin at the level of the external sphincter. She received LcCRT (54 Gy and capecitabine) and on restaging there was no evident clinical response. She underwent laparoscopic total colectomy with abdominoperineal resection and end-ileostomy with resection of a small segment III liver lesion at 11 weeks post-radiotherapy completion (Fig. 1). Histological examination indicated a poorly differentiated ulcerative LCNEC with TRG 2. IHC showed Synaptophysin [+], CD56 [+], Chromogranin A [mostly negative] and Ki67: 80–85% and final staging was ypT3N2bM1a (Table 1). She continued with adjuvant chemotherapy, FOLFOX, until she succumbed to metastatic disease 24 months post-operatively.

## Discussion

Rectal LCNEC is an exceptionally rare and clinically challenging malignancy. Although definition and classification are outlined in the introduction, it is important to reiterate its distinct behaviour and implications. Unfortunately, rectal LCNECs lack clear treatment guidelines, contributing to negative outcomes. Current literature highlights the need for further research to establish treatment protocols [6]. Until then, the significance of IHC in preventing mistreatment cannot be understated. These cases represent the patients which developed the disease before the integration of routine pre-operative IHC. They underscore the importance of IHC as a routine examination for all suspected colorectal tumours to ensure diagnostic accuracy.

Rectal LCNECs are clinically indistinguishable from the common colorectal adenocarcinoma. Symptoms include changes in bowel habits, unexplained weight loss, haematochezia, and tenesmus [3]. The differential includes poorly differentiated adenocarcinoma, small-cell neuroendocrine carcinoma, and mixed neuroendocrine-non-neuroendocrine neoplasms. Evaluating these options requires rigorous histological examination to confirm neuroendocrine markers and large-cell morphological features.

Once a diagnosis is established, treatment should reflect LCNECs aggressive nature. Currently, there are no targeted management protocols for rectal LCNECs, and guidelines are partially based on approaches for high-grade NECs in other organs. According to the 2023 European Neuroendocrine Tumor Society guidelines for the management of digestive neuroendocrine carcinomas, surgical resection is the mainstay treatment before metastatic spread, especially when R0 resection is feasible, and offers a 5-year survival rate of approximately 25%–40% [7]. Furthermore, the guidelines indicate that the efficacy of chemoradiation (CRT) in resectable rectal NECs is unclear; however, most patients likely would have already undergone CRT, given that their tumours were likely assumed to be common adenocarcinoma [7]. Additionally, neo-adjuvant chemotherapy is not deemed necessary but recommended in high-risk cases [7]. Adjuvant chemotherapy, ideally consisting of 4–6 cycles of cisplatin/carboplatin + etoposide, is recommended to combat the high recurrence rates of these neoplasms [7]. This platinum-based regimen is also used in neo-adjuvant settings, whereas in progressive metastatic NECs, 5-FU backbone chemotherapy is an important alternative due to limited confidence in platinum-based options [7]. Surveillance protocols recommend endoscopy, and CT/MRI of the chest, abdomen, and pelvis every 4–6 months for the first two years, then every 6–12 months depending on patient status; however in these presented cases, they were monitored with CT/PET scans every 3 months until death [8, 9]. Unfortunately, prognosis remains poor and due to the delayed diagnosis, often patients will present with advanced disease.

These challenges reiterate the value of further research, specifically for more effective treatment protocols for rectal LCNECs. These two cases highlight the importance of minimizing diagnostic delay via IHC. Mandatory IHC on initial biopsy, now recommended for identifying dMMR cancers, offers the vital collateral benefit of detecting rare but aggressive neoplasms such as LCNEC, preventing misclassification and undertreatment. It is important to raise awareness about these rare cases and encourage multicentre studies to develop treatment standards and prognostic indicators [6]. Perhaps in this new era of omics research, detailed molecular profiling may serve as a steppingstone toward standardized and novel treatments. Until stronger evidence emerges, vigilant use of IHC and diagnostic awareness remain key to managing this rare and challenging malignancy.
